# Mechanistic Insight into Royal Protein Inhibiting the Gram-Positive Bacteria

**DOI:** 10.3390/biom11010064

**Published:** 2021-01-06

**Authors:** Mao Feng, Yu Fang, Chuan Ma, Xiangyuan Duan, Yanyan Zhang, Bin Han, Han Hu, Lifeng Meng, Fuyi Wang, Jianke Li

**Affiliations:** 1 Institute of Apicultural Research/Key Laboratory of Pollinating Insect Biology, Ministry of Agriculture and Rural Affairs, Chinese Academy of Agricultural Sciences, Beijing 100093, China; fengmao@caas.cn (M.F.); fangyu@caas.cn (Y.F.); machuan@caas.cn (C.M.); 82101185168@caas.cn (X.D.); hanbin@caas.cn (B.H.); huhan@caas.cn (H.H.); menglifeng@caas.cn (L.M.); 2 Beijing National Laboratory for Molecular Sciences, CAS Key Laboratory of Analytical Chemistry for Living Biosystems, Institute of Chemistry, Chinese Academy of Sciences, Beijing 100190, China; zhangyy0816@iccas.ac.cn (Y.Z.); fuyi.wang@iccas.ac.cn (F.W.); 3 University of Chinese Academy of Sciences, Beijing 100049, China

**Keywords:** royal jelly, antibacterial, *Paenibacillus larvae*, permeability, cell membrane

## Abstract

Royal jelly (RJ), a natural honeybee product, has a wide range of antibacterial activities. N-glycosylated major royal jelly protein 2 (N-MRJP2), purified from RJ, can inhibit the growth of *Paenibacillus larvae* (*P. larvae*, Gram-positive), a contagious etiological agent of the American foulbrood disease of honeybees. However, the inhibitory mechanism is largely unknown. Antibacterial assay and membrane proteome were conducted to investigate the inhibition capacity of RJ from different instar larvae and *P. larvae* treated by N-MRJP2, respectively. The similar antibacterial efficiency of RJ from different larval instar indicates that RJ is vital for the adaptive immune defense of small larvae. The killing of *P. larvae* by N-MRJP2 is achieved by disturbing the cell wall biosynthesis, increasing the permeability of cell membrane, hindering aerobic respiration, restraining cell division and inducing cell death. This demonstrates that RJ is critical for the passive immunity of immature larvae and N-MRJP2 can be used as natural antibiotic substance to resist *P. larvae*, even for other gram-positive bacteria. This constitutes solid evidence that RJ and N-MRJP2 have potentials as novel antibacterial agents.

## 1. Introduction

American foulbrood (AFB), caused by the spore-forming bacterium *Paenibacillus larvae* subsp. *larvae* (*P. larvae*), is a contagious and lethal disease that threatens honeybees. Honeybee larvae are the most susceptible to be infected during the first two days after hatching, while adults are immune to the infection even if a large number of spores are ingested [1]. Four different genotypes of *P. larvae*, ERIC Ⅰ-Ⅱ, are reported on the basis of the repetitive element PCR using enterobacterial repetitive intergenic consensus (ERIC) sequence primers. ERIC Ⅰ and ERIC Ⅱ are commonly isolated from infected colonies, while ERIC Ⅲ and ERIC Ⅳ are hardly found in clinical identification. The genome sequencing of two *P. larvae* strains, ascribed to ERIC II (DSM 25430) and ERIC I (DSM 25719), reveals the frequent genome rearrangements and a high degree of genome plasticity. This in turn affects the phenotype, including morphology [2], metabolic capacity [3], and most importantly in virulence and virulence factors [4,5]. Exposure bioassays indicate that ERIC Ⅱ is more virulent on the larval level than ERIC Ⅰ. However, ERIC Ⅱ is less virulent on the colony level than ERIC I [6]. A functional S-layer protein (SplA) is a key virulence factor for ERIC Ⅱ, as known from the proteome and gene knockdown analysis [4]. Moreover, Plx1 and Plx2, members of the family of AB toxins that consist of A and B domains, are considered to be virulence factors for ERIC I [5].

AFB is widespread and difficult to control as the spores of *P. larvae* are very resistant to heat, light, and chemicals, and can even remain viable after a long period of dormancy [1]. Although only honeybee larvae are infected and killed by *P. larvae*, the failure to recruit offspring will weaken and even lead to the collapse of the entire colony. Antibiotics, in particular oxytetracycline, are effective for the prevention and treatment of infected colonies. However, oxytetracycline-resistant *P. larvae* have been isolated in Argentina and the United States [7]. Furthermore, the application of antibiotics could diminish the lifespan of honeybees, cause disequilibria in the normal microbiota of the hive, and increase the risk of bee product contamination by drug residues. In some European countries, the diseased colonies have to be burnt because the application of antibiotics in beekeeping is illegal [8]. This causes not only economic losses for the beekeeping industry, but also a threat to plants that depend on bee pollination. Using phages to infect and lyse *P. larvae* is a potential treatment to control AFB, even though no deleterious effect has been found on artificially reared honeybee larvae [9], testing under in-hive conditions is still needed. So, the search for natural biocides for the control of AFB is urgent and with great challenge.

Royal jelly (RJ), a proteinaceous secretion synthesized and secreted by the hypopharyngeal glands and mandibular glands of nurse bees, plays vital roles in honeybee nutrition, caste differentiation, and is health-promoting for human beings. It serves as the primary nutrition for larvae during the first three days after hatching and the exclusive food for queens throughout their lifetime. Continuous feeding of ample RJ amounts causes a rapid development of the larvae into queens with a large body size, fully matured ovaries, and a longer life span. In contrast, larvae that are fed with RJ only during the first three days develop into short-lived and functionally-sterile worker bees, which is known as caste determination [10]. Moreover, a wide cascade of health-promoting properties has been reported, such as anti-bacterial [11], anti-inflammatory [12], anti-oxidative [13], anti-fatigue [14], anti-hypertensive [15], antitumor [16], and anti-diabetic capacities [17]. Furthermore, RJ exhibits neurotrophic effects that improve the spatial memory performance of mice suffering from Alzheimer’s disease [18] and facilitate mice with a damaged hippocampal dentate gyrus to regain cognitive abilities [19]. Major royal jelly proteins (MRJP1), the main component of RJ proteins, may improve spatial memory via the cysteine and taurine metabolism and energy metabolism pathways in aged rats [20]. To date, RJ is commonly used in pharmacological, cosmetics, food, and nutraceutical industries [21].

RJ and its derivate components also exhibit a broad spectrum of antimicrobial activities [11,22]. Royalisin, an insect defensin in RJ, could fight against fungi, and Gram-positive and Gram-negative bacteria [22]. The fatty acid 10-hydroxy-2-decenoic acid (10-HDA), unique to RJ, plays a role in antimicrobial activities of RJ as well [23]. Jelleines, a family of antimicrobial peptides derived from the tryptic digested MRJP1, also have a wide range of activity in resistance against Gram-positive and Gram-negative bacteria, and yeasts [24]. Additionally, proteins are essential factors for RJ to achieve this antibacterial capacity. Glucose oxidase in RJ has a high antibacterial activity on the vegetative form of *P. larvae* (ATCC9545) [25]. Apolipophorin III-like protein from *Apis Cerana* possesses antibacterial activities by binding to the Gram-positive and Gram-negative bacterial cell wall components [26]. Interestingly, the glycosylated MRJP1 from honey could fight against two clinically isolated multi-drug resistant bacteria: vancomycin-resistant *Enterococci* and methicillin-resistant *Staphylococcus aureus* [27]. Purified N-glycosylated MRJP2 (N-MRJP2) from RJ could inhibit *P. larvae*, whereas the deglycosylated form could not using the minimum inhibitory concentration method [28]. MRJP2 harbors 17 N-glycans in the three N-glycosylation sites [29] with a molecular mass from 50 to 60 kDa [30]. However, the inhibitory mechanism of N-MRJP2 against *P. larvae* is still elusive.

RJ is produced by grafting young larvae from worker cells into queen cells to induce nurse bees to provision larvae with RJ. Then, it is harvested during 68–72 h (about third instar) after larval transfer, which is the time window that the maximum amount of RJ accumulation reaches [31]. However, RJ can also be harvested earlier during the first and second instar. Despite the concentrations of 10-HDA [32], free amino acids, total amino acids [33], and anti-oxidant activity [34] in RJ are varied with the grafted larval ages, the antibacterial differences between RJ fed to the first, second and third instar larvae are largely unknown. As previously reported, N-MRJP2 inhibited the growth of *P. larvae* [28], a property that has great potential in controlling the lethal honeybee disease caused by contiguous *P. larvae*. The mechanistic insight into *P. larvae* inhibition by N-MRJP2 however still needs to be recovered. Therefore, membrane proteome was investigated to reveal how *P. larvae* is inhibited by N-MRJP2 because MRJPs are ascribable to the antimicrobial peptide (AMP) category, and the classic mechanism of AMP is to disrupt the microbial plasma membrane [35]. This will provide a novel molecular understanding of the N-MRJP2 resistance against *P. larvae*, and may potentially be useful in the control of AFB and other gram-positive bacteria as a natural antimicrobial.

## 2. Materials and Methods

### 2.1. Sampling of RJ from the First, Second, and Third Instar Larvae

Honeybees (*Apis mellifera ligustica*) were kept in the apiary of the Institute of Apicultural Research, Chinese Academy of Agricultural Sciences, Beijing, China. Nine colonies with identical honeybee populations, food storage, and brood pattern, each headed by an egg-laying queen, were assigned randomly to 3 groups. To ensure the known age of the larvae for grafting, the honeybee queen was confined to an empty worker comb frame with a queen excluder for 4 h, through which workers but not the queen could pass. Subsequently, the queens were removed and the frames containing the laid eggs were maintained in the colonies for hatching. RJ frames with 3 strips of plastic queen cell cups (33 cells/strip) were applied in each colony. The frames containing the grafted worker larvae (less than 24 h old after hatching) were put into the colonies, of which only one strip of plastic queen cell cups in each colony was sequentially withdrawn after 24, 48, and 72 h for RJ collection.

### 2.2. Antibacterial Assays of RJ Aqueous Solution, Water-Soluble Fractions, and N-MRJP2

Two Gram-negative bacteria: *Escherichia coli* (*E. coli*, ATCC 25922) and *Pseudomonas aeruginosa* (*P. aeruginosa*, ATCC 27853), and three Gram-positive bacteria: *Bacillus subtilis* (*B. subtilis*, ATCC 21332), *Staphylococcus aureus* (*S. aureus*, ATCC 6538), and *P. larvae* were applied to determine the antibacterial activity of RJ related to different aged larvae. This strain of *P. larvae* is purchased from the China General Microbiological Culture Collection Center, however, the genotype of which is unclear. RJ aqueous solution, water-soluble RJ proteins, and purified N-MRJP2 were determined using oxford cup assay. *E. coli*, *P. aeruginosa*, *B. subtilis* and *S. aureus* were cultured with Luria-Bertani medium (10 g/L tryptone, 5 g/L yeast extract, 10 g/L NaCl, pH 7.4), while *P. larvae* was cultured with MYPGP media (1% Mueller-Hinton broth, 1.5% yeast extract, 0.3% K_2_HPO_4_, 0.1% Na-pyruvate and 0.2% glucose). In brief, 1 mL of bacterial culture solution with almost 1 × 10^8^ colony-forming unit was mixed with 200 mL agar medium (medium with 2% agar) and then cast in petri dishes. The RJ aqueous solution was prepared with ddH_2_O and then homogenized with an ultrasonic wave at the concentration of 5%, 10%, 20%, 40%, 60%, and 80% (*w*/*w*), respectively. Water-soluble RJ protein and N-MRJP2 were prepared based on the previously described methods with some modification [28,36]. In brief, RJ was dissolved in 40 mM phosphate buffer (pH 8.0) (1:10, *w*/*v*). The suspension was centrifuged at 25,000× *g*, for 15 min at 4 °C. Then the supernatant was collected and filtered with the 0.45 μm membrane filter, followed by 30 kDa MM cutoff by using centrifugal filter unit (Millipore, Billerica, MA, USA). The solution of flowing-through was discarded. The remainder was further purified by the Q-Sepharose (GE Healthcare, Piscataway, NJ, USA). The protein was eluted with a linear gradient of NaCl from 0 to 1.0 M at a flow rate of 0.5 mL/min. Fractions with single elution peak were collected and detected by SDS-PAGE, those with single band were taken as purified protein candidates. The eluted protein was considered as MRJP2 after identification by MS/MS. The identified MRJP2 was then treated with PNGase F, which can effectively remove the attached N-glycans. Those were confirmed as N-MRJP2 only if the untreated MRJP2 showed a single band, and the treated MRJP2 exhibited a single band that was a little lower than that of the untreated one visualized by SDS-PAGE, because the increasing of the electrophoretic mobility of a protein after treatment with glycosidases provides a convenient way to understand which types of glycans are present in a protein of interest [37].

The resulting solutions were added to the Oxford cups which were placed at equal distances above the agar surfaces. The plates were photographed and the diameter of each inhibition zone was measured after 4 h of incubation at 37 °C (30 °C for *P. larvae*). The procedures were produced in triplicate.

### 2.3. Genotyping of P. larvae

To ensure the genotype of the tested *P. larvae*, the bacterial DNA for fingerprinting was prepared and subsequent rep-PCR analysis was performed as previously described [38]. In brief, the DNA of *P. larvae* were isolated using 6% InstaGene matrix (Bio-Rad) following the manufacturer’s instructions. The DNA sequences of the primers used for rep-PCR were ERIC1R-ATGTAAGCTCCTGGGGATTCAC and ERIC2-AAGTAAGTGACTGGGGTGAGCG. PCR was carried out in a final volume of 25 μL consisting of 1 × reaction buffer (Qiagen, Germantown, MD, USA) and final concentrations of 2.5 mM MgCl_2_, 250 mM dNTPs, 10 mM primer, and 0.3 U HotStarTaq polymerase (Qiagen). The reaction conditions were as follows: after the initial activation step (15 min, 95 °C); 35 cycles at 94 °C for 1 min, 53 °C for 1 min, and 72 °C for 3 min, followed by a final elongation step at 72 °C for 10 min. A 10 μL sample from the PCR was analyzed on a 0.8% agarose gel. DNA bands were stained with ethidium bromide and visualized under UV light. Then the genotype of *P. Larvae* applied in this investigation could be discriminated by the comparison between the resulted DNA bands and the genotype-specific DNA bands supplied by Genersch et al. (2006) [2].

### 2.4. Determination of Released UV-Absorbing Material of P. larvae

Cell membrane damage of *P. larvae* was evaluated by determining the leakage of intracellular material absorbing at 260 nm after the treatment with N-MRJP2. Optical density (OD) value at 600 nm of overnight cultured *P. larvae* in MYPGP medium was adjusted to 1.0. Cells were harvested by centrifugation at 400× *g* for 15 min. The supernatant was discarded, and then the pellet was washed with sterile peptone H_2_O and centrifuged twice, and finally suspended in sterile H_2_O. Before the treatment, a microdilution assay was used to determine the minimum inhibitory concentration (MIC) of N-MRJP2 against *P. larvae* according to the previously published method [28]. N-MRJP2 was added to the cell suspension at the MIC level (16 μg/μL). Control samples were prepared similarly without adding N-MRJP2. The samples were then incubated at 30 °C for 60 min. Afterwards, the cell suspension was centrifuged at 13,500× *g* for 15 min, and the OD at 260 nm of the supernatant was measured [39,40]. The released UV-absorbing materials were represented by the mean values ± SD by measuring three independent replicates.

### 2.5. Intracellular Ca^2+^ Concentration Evaluation by Laser Confocal Microscopy and Time-of-Flight Secondary Ion Mass Spectrometry (ToF-SIMS)

To assess the intracellular Ca^2+^ concentration, the N-MRJP2 treated *P. larvae* and its control were incubated with Fluo 3-AM (Solarbio, Beijing, China), a membrane permeable fluorescent probe used to detect the changes in intracellular calcium level, at 30 °C for 60 min according to the manufacturer’s instruction. Fluorescence was monitored at 488 nm (emission wavelength: 525–530 nm) using a confocal laser microscope system (X81, Olympus, Japan). *P. larvae* with and without N-MRJP2 were taken as treated and control group. Briefly, 512 by 512 pixels x-y images were designed, and aeras with comparable bacterial amount were chosen to generate the target images. The acquired images were analyzed with Image J software to get the fluorescence intensity based on the entire image (1.48 V, NIH, Bethesda, MD, USA).

ToF-SIMS was applied to further investigate the changes of intracellular Ca^2+^ under N-MRJP2 treatment within a single bacterial cell. The cultured *P. larvae* were collected and centrifuged at 4000× *g* for 10 min. The precipitate was kept and washed with phosphate buffer saline (PBS) buffer for three times, followed by 150 mM ammonium acetate, then centrifuged at 8000× *g* for 2 min. The well-washed bacteria were then diluted with 150 mM ammonium acetate and the OD 600 value was adjusted to 0.2~0.3. A 2 mL sample of bacterial solution was directly drop-coated onto a pre-prepared silicon wafer. The samples were frozen with liquid nitrogen and lyophilized for ToF-SIMS analysis. ToF-SIMS experiments were performed on a ToF-SIMS V instrument (Ion-TOF GmbH, Münster, Germany). During depth profiling, a 30 keV pulsed Bi_3_^+^ liquid metal ion gun was used for analysis beam and 5 keV Ar_1800_^+^ gas cluster ion beam was used for sputtering materials away. The beam repeating frequency was 10 kHz. The current of the analysis beam was ~0.8 pA in the high current bunched mode, and that of the sputter beam was adjusted to ~0.5 nA with a lead off value of 52.8 µs. The raster area of the analysis beam was 100 × 100 µm^2^ and that of the sputter beam was 300 × 300 µm^2^. The measurements were stopped when *P. larvae* in the crater of the sputter beam were completely sputtered away. For ToF-SIMS imaging experiments, data were collected by using the pulsed Bi_3_^+^ analysis beam with a current of 0.09 pA by 256 × 256 pixels. The raster size of it was 100 × 100 µm^2^. The beam was operated in ultimate imaging mode and delay extraction mode was applied for improved mass resolution. At first, the sputter gun was used with a current of ~0.5 nA and a raster size of 500 × 500 µm^2^ to clean possible surface contamination for 40 scans. Then, a lower current of ~0.2 nA and a raster size of 300 × 300 µm^2^ was used for ToF-SIMS images collection. During all ToF-SIMS measurements, a low energy electron gun with a current of ~2.2 µA was used for charge compensation.

### 2.6. Membrane Protein Preparation of P. larvae

Bacterial cell membranes are possible targets for the development of antibacterial agents [41], so the membrane proteome of *P. larvae* was investigated here.

*P. larvae* were incubated at 30 °C in MYPGP media at a concentration of 1 × 10^6^ colony-forming units/mL. Purified N-MRJP2 were serially diluted and added to the medium at the MIC level (16 μg/μL), then incubated for another 60 min. Then the *P. larvae* spores were harvested by centrifugation and washed with PBS six times. Membrane proteins were prepared as described [42]. In brief, the purified spores were treated with decoating solution (0.1 M NaCl, 0.1 M NaOH, 1% sodium dodecyl sulfate, 0.1 M DTT) at 70 °C for 1 h. After intensive washing with water, the decoated spores were incubated with TEP buffer (50 mM Tris-HCl [pH 7.4], 5 mM EDTA, 1 mM phenylmethylsulfonyl fluoride) containing 2 mg/mL lysozyme and 40 μg/mL MgCl_2_ at 37 °C for 30 min, and subsequently smashed with an ultrasonic cell disruptor (power: 180 W, working time: 5 s, intermittent time: 10 s, repeating for: 30 times; Scientz, Ningbo, China) on ice. Then, the suspension was centrifuged at 15,000× *g* for 10 min at 4 °C to remove unbroken cells and integuments. NaCl was added at a final concentration of 1 M to the collected supernatant, which contained the membrane and soluble fractions. The solution was centrifuged at 100,000× *g* for 1 h at 4 °C. The pellet was kept and washed to retrieve the membrane proteins. Then the pellet was dissolved, reduced, alkylated, and digested by trypsin as described above.

### 2.7. Membrane Proteome Analysis of P. larvae

The final concentration of digested peptides was determined by UV-spectrometry (Nanodrop, Thermo) using an extinction coefficient of 1.1 for 0.1% (g/L) solution at 280 nm. The prepared peptide samples of *P. larvae* were analyzed using a Q Exactive HF system (Thermo Fisher Scientific, Waltham, MA, USA) coupled to an Easy-nLC 1000 (Thermo Fisher Scientific) via a nanoelectrospray ion source. Samples were loaded onto a fused silica trap column containing 5.0 μm Aqua C18 beads (100 μm × 20 mm, Thermo Fisher Scientific) for 2 min in buffer A (0.1% formic acid) at a flow rate of 5 μL/min. Peptides were separated with a fused silica analytical column (75 μm × 150 mm) filled with 3 μm Aqua C18 beads (100 Å, Thermo Fisher Scientific) at a flow rate of 350 nL/min. The elution gradient was as follows: from 3 to 8% buffer B (0.1% formic acid in acetonitrile) for 10 min, from 8 to 23% buffer B for 130 min, from 23 to 30% buffer B for 20 min, from 30 to 90% buffer B for 8 min, and 90% buffer B for 12 min. A data-dependent acquisition mode was applied for full MS scans ranging from *m*/*z* 300–1800 with a resolution of 70,000; the top 15 most abundant precursor ions were fragmented by higher energy collisional dissociation with a resolution of 17,500 and started from *m*/*z* 100, isolation window 2 *m*/*z*, with normalized collision energy of 27. The dynamical exclusion setting was as follows: charge exclusion, unassigned 1, >8; peptide match, preferred; exclude isotopes, on; dynamic exclusion, 10 s. The MS/MS data were acquired in raw files using the Xcalibur software (version 2.2, Thermo Fisher Scientific).

### 2.8. Label-Free Quantification of Abundant Level of Membrane Protein

To identify the proteins of *P. larvae*, the MS/MS data were searched against a combined database containing protein sequences of *P. larvae* (8100 entries, downloaded in September, 2016 from NCBI) and a repository of adventitious proteins database, cRAP, by using in-house PEAKS software (version 8.0, Bioinformatics Solutions Inc, Waterloo, ON, Canada). The search parameters were as follows: enzyme, trypsin; precursor mass tolerances, 20 ppm; fragment mass tolerances, 0.05 Da; allowing a nonspecific cleavage, maximum missed cleavages per peptide, 2; maximum allowed variable PTM per peptide, 3; fixed modification, carbamidomethyl (C, +57.02); variable modifications, oxidation (M, +15.99). A fusion-decoy database searching strategy, which is more conservative in false discovery rate (FDR) estimation than a target-decoy approach, was applied and a protein was considered to be confidently identified only if it had at least two unique peptides when applying a threshold of FDR ≤1.0%.

Relative protein abundance levels were quantified by the label-free approach using PEAKS Q module (version 8.0 Bioinformatics Solutions Inc. Waterloo, ON, Canada). Each sample was submitted in triplicate to PEAKS and one of them was automatically selected as a reference by the software. Feature detection was performed based on an expectation-maximization algorithm, while the features of the same peptides from different samples were aligned through a high-performance retention time alignment algorithm. Total iron current was used to normalize the sample intensity during quantification. Protein expressions of all samples were quantified with the sum of three of the most abundant precursor ion peak intensities of the tryptic peptides. Proteins from different samples were considered to be significantly changed in their abundance levels only with a *p* value <0.05 and a fold change of ≥1.5.

### 2.9. Bioinformatics Analysis of Membrane Proteome

Membrane proteins were assessed based on the combination of transmembrane domain or signal peptide prediction by applying the following on-line programs: TMHMM (http://www.cbs.dtu.dk/services/TMHMM/) [43], Phobius (http://phobius.sbc.su.se/) [44], PSORT v3.0.2 (http://www.psort.org/psortb/) [45], PRED-LIPO (http://bioinformatics.biol.uoa.gr/PRED-LIPO/index.jsp) [46] and Inter-ProScan 5 (http://www.ebi.ac.uk/interpro/) [47]. Proteins were considered as membrane proteins when at least three out of the five were predicted. To gain functional insight of identified membrane proteins, SubtiWiki, a database for the model organism *B. subtilis* that links pathway, interaction, and expression information [48], was applied based on the *P. larvae* sequence alignment using local BlastP against *B. subtilis* database downloaded from UniProt with the e-value <10^−10^.

The gene ontology (GO) enrichment was performed using ClueGO (version 2.2.3), a Cytoscape (version 3.2.1) plugin, by comparing the input data set with the entire genome of *B. subtilis* as the background. The enriched GO terms in biological processes were reported using a right-sided hyper-geometric test. Then, Bonferroni step-down was used to correct the *p*-value to control FDR, and only GO terms with *p* ≤ 0.05 were considered significantly enriched.

### 2.10. Western Blotting Verification of Protein Related to Chemotaxis

Western blot analysis was performed to verify the expression level of methyl-accepting chemotaxis protein (McpC), a chemoreceptor in chemotaxis, of *P. larvae* after the treatment with N-MRJP2. The total amount of protein was extracted from cells, and the concentration was determined by Bradford Kit (Sangon Biotech, Shanghai, China). Equal amounts of proteins were then separated by stacking (4%) and separating (12%) SDS-PAGE, followed by transfer to polyvinylidene difluoride (PVDF) membranes (Invitrogen). After being blocked with 5% skimmed milk in Tris-buffered saline with Tween (TBST) 20 for 1 h, the membrane was washed and incubated with a primary antibody of McpC (Signalway antibody, ShenYang, China) at a dilution of 1:2000, then washed with TBST three times and incubated with secondary antibodies (goat anti-mouse, Invitrogen) at room temperature for 1 h. The blots were visualized using the Chemic Doc XRS imaging system (Bio-Rad) in the presence of enhanced chemiluminescence (Pierce, Thermo Fisher). The targeted protein band was quantified by NIH Image J software and normalized by Sodium Potassium ATPase, which was used as a loading control and its antibody was purchased from Abcam (Cambridge, MA, USA).

### 2.11. Statistical Analysis

All experiments were performed at least with three replicates. The statistical analysis was performed by independent samples t-test or one-way ANOVA analysis (for label-free proteomic quantification) using SPSS (version 16.0, SPSS, Inc. Chicago, IL, USA). A probability level of 5% (*p* < 0.05) was considered to be significant.

## 3. Results

### 3.1. A Similar Antibacterial Activity of RJ from Queen Cells of Immature Instar Larvae

**Figure 1:**
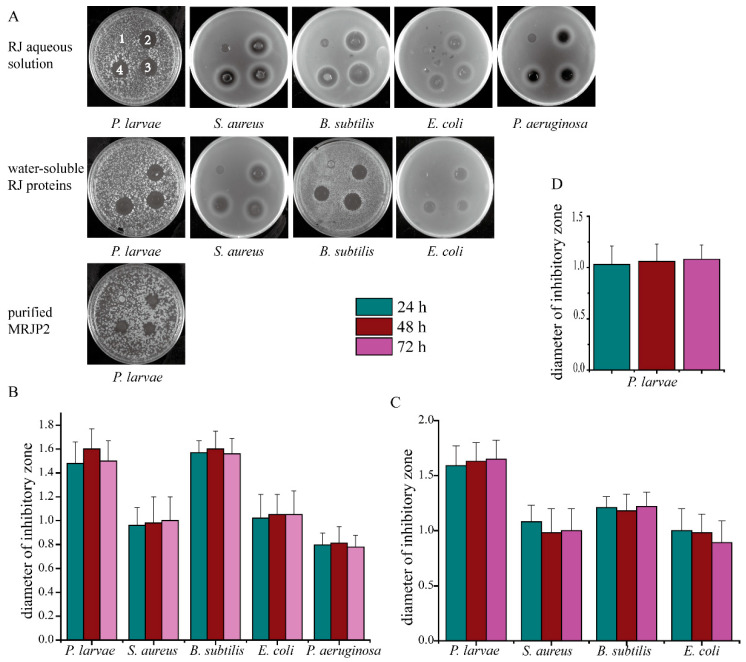
Inhibition plates of royal jelly (RJ) and its derivate components. RJ was collected at 24, 48 and 72 h after the honeybee larvae transferred into the queen cells. (**A**): Inhibition plates of RJ aqueous solution (10%, *w*/*w*), water-soluble RJ fractions, and purified N-glycosyalted major royal jelly protein 2 (N-MRJP2) against *Paenibacillus larvae* (*P. larvae*), *Staphylococcus aureus* (*S. aureus*), *Bacillus subtilis* (*B. subtilis*), *Escherichia coli* (*E. coli*), and *Pseudomonas aeruginosa* (*P. aeruginosa*), respectively. “1”, “2”, “3” and “4” represent the blank control, solution from RJ harvested at 24, 48, and 72 h after larvae transfer, respectively. (**B**–**D**) represent the diameter of inhibitory zones of different bacterial strains treatment with RJ aqueous solution (10%, *w*/*w*), water-soluble RJ fractions (10 μg/μL), and purified N-MRJP2, respectively.

Three common food-borne pathogens: *E. coli*, *P. aeruginosa* and *S. aureus*, and two other Gram-positive bacteria: *B. subtilis* and *P. larvae*, were used for antibacterial assay. It was obvious that these five bacterial strains can be effectively inhibited by 10% aqueous RJ solution from RJ of the first, second and third instar larvae, as revealed by the apparent inhibitory zones, respectively (Figure 1A). Moreover, no differences were found among the diameter of inhibitory zones after the comparison of the RJ harvested at different larval instar on each bacterial strain (Figure 1B). Furthermore, the antibacterial capacity was positively correlated with the concentration of RJ aqueous solution compared to the blank controls (Figure S1).

Evident inhibitory zones were also observed when *E. coli*, *B. subtilis*, *S. aureus* and *P. larvae* were treated with the water-soluble fractions (10 μg/μL) of RJ except *P. aeruginosa*, however, no significant differences among the diameter of the inhibitory zones were observed with respect to each bacterial strain (Figure 1C). To be noted, for the purified N-MRJP2, no inhibitory activity was observed for the tested bacteria except for *P. larvae*, which showed no significant difference in inhibitory zones of RJ from different larval instars (Figure 1D).

### 3.2. Genotype Pattern of P. larvae Using Specific Gel Bands Acquired by Rep-PCR

**Figure 2:**
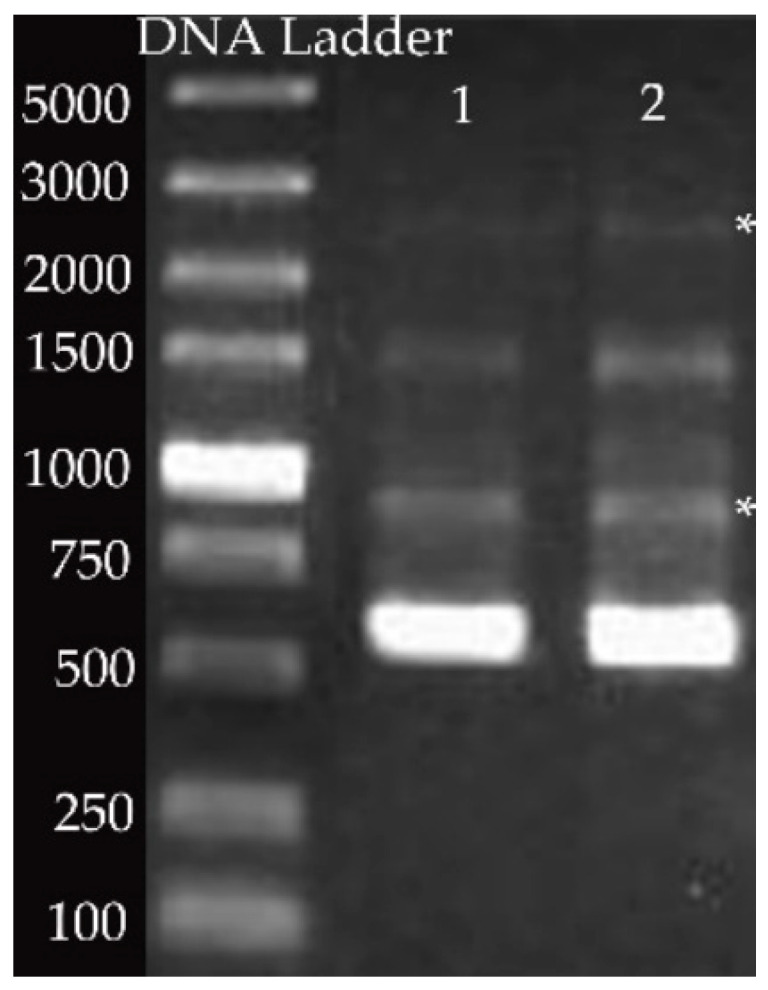
Genotyping analysis via rep-PCR uses ERIC primers. Using the primer pair ERIC1R/ERIC2, strain of *Paenibacillus larvae* (*P. larvae*) was analyzed. The left band is the DNA ladder (bp), the right two bands (“1” and “2”) are the replicates of *P. larvae*. The bands migrating at 970 bp and 2800 bp, considered specific for ERIC Ⅱ, are highlighted by asterisks.

Based on PCR results using ERIC primers, several bands were resolved in the gel. Most notably, a band of 970 bp and an additional band migrating at 2800 bp, considered specific for ERIC Ⅱ, were visualized (Figure 2).

### 3.3. N-MRJP2 Treatment Inducing a Cell Membrane Permeability Increase in P. larvae

**Figure 3:**
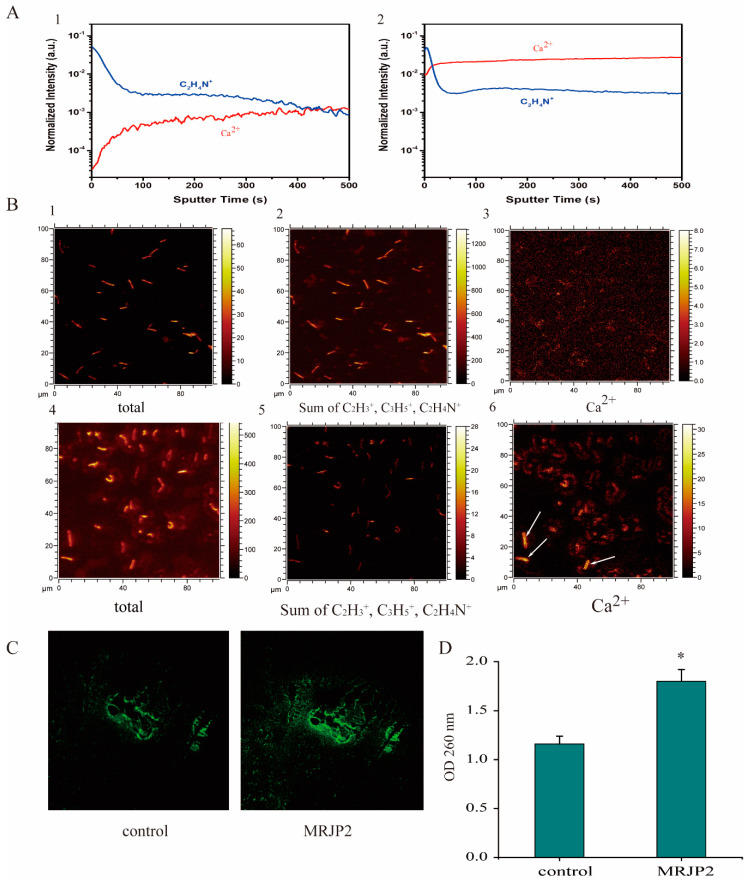
Intracellular Ca^2+^ intensity and UV absorbance measurement of *Paenibacillus larvae* (*P. larvae*). (**A**): Depth profiling of *P. larvae* by time-of-flight secondary ion mass spectrometry (ToF-SIMS) before (1) and after (2) treatment with N-glycosylated major royal jelly protein 2 (N-MRJP2). Ion intensities were normalized to total counts. (**B**): ToF-SIMS images of *P. larvae* before (1–3) and after (4–6) N-MRJP2 treatment. (1, 4), total ion images; (2, 5), images of the sum ion intensity of C_2_H_3_+ (*m*/*z* 27), C_3_H_5_+ (*m*/*z* 41) and C_2_H_4_N+ (*m*/*z* 42) and (3, 6) Ca^2+^ ion images, cells with relatively stronger ion intensity of Ca^2+^ were shown by arrows. (**C**): Representative fluorescence images of intracellular Ca^2+^ concentration evaluated with Fluo-3/AM probe. (**D**): UV absorbance measurement of *P. larvae* before (control) and after (MRJP2) treatment with N-MRJP2. Optical density (OD) at 260 nm was observed. “*” demonstrates significant differences compared to control (*p <* 0.05).

Based on the in-depth profiling of *P. larvae* using time-of-flight secondary ion mass spectrometry (ToF-SIMS), the signal intensity of C_2_H_4_N^+^ ion (*m*/*z* 42) of N-MRJP2 treated *P. larvae*, a fragment ion of organic species in *P. larvae*, was similar to that in the untreated sample, and can be used as the reference ion to estimate the variation of the system and to normalize the ion intensity of Ca^2+^ (Figure 3(A1,A2)). However, the ion intensity of Ca^2+^ (*m*/*z* 40) was significantly enhanced by >10 folds after N-MRJP2 treatment (Figure 3A). In order to demonstrate the effect of the N-MRJP2 treatment on the uptake of Ca^2+^ into the bacteria, ToF-SIMS imaging was also applied to investigate the changes of Ca^2+^ intensity inside the bacteria before and after treatment at a single cell level of *P. larvae*. Total ions intensity and the sum ion intensity of C_2_H_3_+ (*m*/*z* 27), C_3_H_5_+ (*m*/*z* 41), and C_2_H_4_N+ (*m*/*z* 42) was used to render clear images of *P. larvae*. In the untreated sample, the Ca^2+^ intensity in each bacterial cell was very low (Figure 3(B3)). However, after treatment, obvious distributions of Ca^2+^ inside each cell were observed (brilliant yellow, Figure 3(B4–B6)), the intensity of which was significantly elevated in comparison with that of the untreated cells. Moreover, a relatively stronger ion intensity of Ca^2+^ was found for some cells (approximatively 12% of the population) as shown by arrows in Figure 3(B6).

The intensity levels of Ca^2+^ was monitored with the fluorescent probe Fluo-3/AM using a confocal fluorescence microscope. The fluorescent intensity (FI, green colored) was quite weak in the control group, but was remarkably elevated in the treatment group (FI_treatment_:FI_control_ = 1.51) (Figure 3C).

The light absorbance of *P. larvae* medium at 260 nm in the presence of N-MRJP2 increased significantly compared to the control (Figure 3D), which indicates that the N-MRJP2 treatment can result in the leakage of the intracellular DNA and RNA.

### 3.4. Membrane Proteome Dissecting Molecular Basis of N-MRJP2 against P. larvae

**Figure 4:**
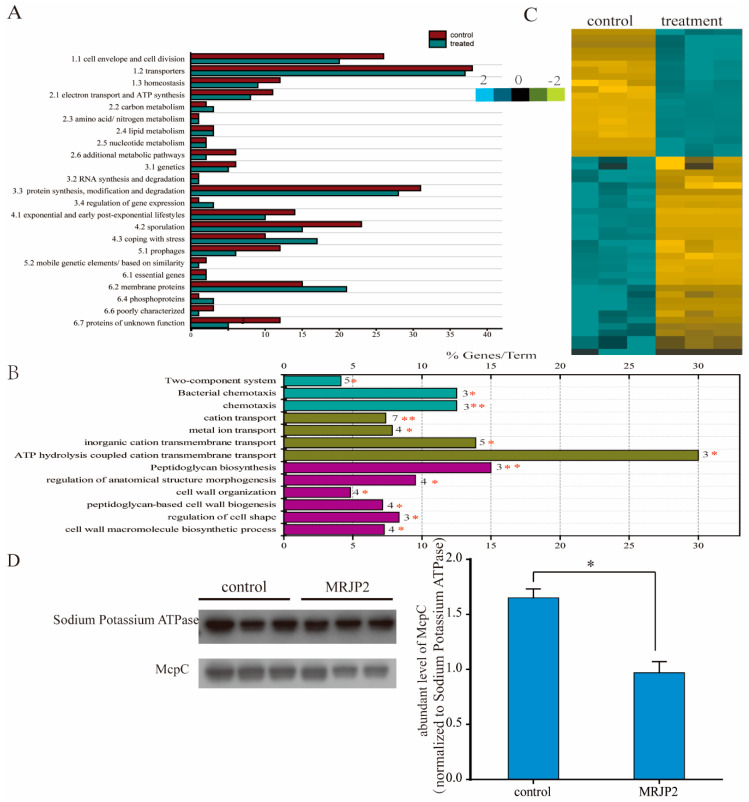
Qualitative and quantitative comparison of membrane proteins of *Paenibacillus larvae* before and after treatment with N-glycosylated major royal jelly protein 2 (N-MRJP2). (**A**) Categorization of proteins according to SubtiWiki based on the sequence alignment using BlastP against *Bacillus subtilis* database downloaded from UniProt with the e-value < 1.0 × 10^−10^. (**B**) Enriched functional groups of the differentially expressed membrane proteins. % genes/Term represents the proportion of genes enriched in corresponding functional groups. The bars with the same color represent they belong to the same functional group. The numbers stand for the genes enriched to the corresponding functional group. “*”, *p* < 0.05; “**”, *p* < 0.01. (**C**) Unsupervised hierarchical clustering of the differentially expressed membrane proteins before and after the treatment with N-MRJP2 (fold changes > 2, *p <* 0.05). The high- and low-intensity level of membrane proteins are indicated by light blue and darkgolden color code. (**D**) Western blot analysis of the relative expression level (fold change) of methyl-accepting chemotaxis protein (McpC) before (control) and after (MRJP2) the treatment with N-glycosylated MRJP2. Sodium potassium ATPase is used as reference control.

In total, 1244 and 1287 protein groups including 1913 and 1980 proteins were identified in *P. larvae* of the control and treated samples, respectively. Only 205 protein groups (311 proteins) and 216 protein groups (331 proteins) were identified as membrane proteins after filtration (Table S1). Based on the functional categorization of SubtiWiki, the control and the N-MRJP2 treated *P. larvae* shared all of the 23 functional groups in addition to some groups that were implemented by a different set of proteins. However, in many groups the number of identified proteins in the control was higher than that of the N-MRJP2 treated samples. For instance, 26 proteins involved in cell envelope and cell division were identified in the control group, whereas only 20 were found in the treated group. In the control, proteins related to cell envelope and cell division, homeostasis, electron transport and ATP synthesis, protein synthesis/modification/degradation, exponential and early post-exponential lifestyles, sporulation and germination, and prophages categories were overrepresented. In N-MRJP2 treated groups, proteins involved in carbon metabolism, gene expression regulation, coping with stress, and proteins acting as transporter were overrepresented. Only five groups: amino acid/nitrogen metabolism, lipid metabolism, nucleotide metabolism, RNA synthesis and degradation, and essential genes, were similarly represented in both sample groups (Figure 4A).

Of all the membrane proteins, only 51 proteins in 50 protein groups were differentially regulated. They were enriched in three biological process: peptidoglycan biosynthesis (dacF, murG and pbpF), bacterial chemotaxis (McpC, motA and tlpA), and cation transport (copA, cydC, motA, qoxA, ydfM, yloB and zosA) (Figure 4B). Most of the 51 proteins were significantly down-regulated after the treatment with N-MRJP2 (Figure 4C). Western blotting confirmed that the abundance level of McpC, which is involved in the regulation of chemotaxis, was remarkably reduced when N-MRJP2 was applied (Figure 4D), in accordance with the proteome data.

## 4. Discussion

To gain a comprehensive novel knowledge of the antibacterial capacity of RJ and the inhibitory effects of N-MRJP2 on *P. larvae*, antibacterial effects of RJ from the different instar larvae and membrane proteome of *P. larvae* treated with N-MRJP2 were analyzed. The comparative antibacterial activity of RJ, and the equivalent bacteriostatic effect of its water-soluble fractions from different honeybee larvae instar indicate that RJ is critical for the young larval immune defense and bee health regulation. Moreover, the inhibitory activity of N- MRJP2 on *P. larvae* is associated with the increasing the permeability of cell membrane, blocking the synthesis of cell wall, and eventually hindering respiration and inducing programed cell death (PCD).

### 4.1. Immune Regulatory Capacity of RJ to Ensure Larval Development

Passive immune response is a necessary and useful supplement to honeybee larvae, especially for small larvae (from the first and second instar) [49], whose innate immune system is not matured yet [50]. The effective inhibitory capacity of aqueous solution of RJ against *B. subtilis*, *S. aureus*, *E. coli*, *P. aeruginosa*, and *P. larvae* reflected by the agar disk-diffusion assay, which is commonly used for antimicrobial screening [51], may be achieved by RJ and its derivate components, such as royalisin, 10-HDA, jelleines, apismin and MRJPs that could resist against a wide range of microorganisms including Gram-positive and Gram-negative bacteria [11]. The antibacterial effects of the water-soluble fraction against *B. subtilis*, *S. aureus*, *E. coli*, and *P. larvae* are likely due to the existence of the royalisin, MRJPs, and other components. As an amphipathic protein, royalisin has antibacterial activity against Gram-positive and Gram-negative bacteria at low concentrations, such as *B. subtilis*, *S. aureus* and *E. coli* [22]. Here, a Gram-negative bacteria strain, *P. aeruginosa*, was not inhibited by the RJ water-soluble fraction, which is in line with finding that the hydrosoluble fraction of RJ is more effective to Gram-positive bacteria [52]. Here, in addition to a similar inhibitory activity of RJ aqueous solution, the sensitive of the water-soluble ingredients of the RJ samples from three larval instars to different bacterial strains indicate that RJ and its active components are crucial for the honeybee’s larval passive immunity. This is of paramount importance for small larvae whose innate system is not mature enough to provide effective protection during larval development [50].

Although similar antibacterial activities of RJ grafted from different instar larvae were shown, the high susceptibility of small honeybee larvae (around 24 h after egg hatching) to *P. larvae* might be caused by (i) the innate immune system of young larvae being much weaker than that of the old larvae because the innate immune response of honeybee evolves across the developmental stages [50]; (ii) the smaller amount of RJ supply to small larvae. Insects have diverse ways to combat infection by pathogens beyond innate immune response. Many insects are protected by a layer of antimicrobial secretions on their exterior, and by a gut environment that is hostile to pathogens [53]. Small larvae are provided with less RJ than that of old larvae during the nursing period, so the RJ accumulation in comb cells and the ingestion of RJ into the larval intestinal tracts are not adequate enough to yield sufficient passive immune protection against the invaded *P. larvae* given that RJ inhibits *P. larvae* in a dose-dependent manner [28].

### 4.2. N-MRJP2 Has Potential to Kill P. larvae of ERIC II Pattern

Genotyping of *P. larvae* strains show four different ERIC patterns, ERIC I-ERIC IV, which is classified based on DNA fingerprinting. The ERIC I and II genotypes of *P. larvae* possess a specific band migration at about 970 bp, but differed in one prominent band migration at about 2800 bp and in one minor band at about 1200 bp. ERIC III is confirmed with a double band migration between 1500 and 2000 bp and ERIC IV is characterized by having no band migrating greater than 1250 bp [2]. Here, the reference bands migrating at around 970 bp and 2800 bp indicate that the genotype of *P. larvae* used is ERIC II and N-MRJP2 can inhibit *P. larvae* of ERIC II pattern. Further testing is needed to make sure whether N-MRJP2 is also effective for the other three genotypes due to the different spore morphology and virulence among them [6].

### 4.3. N-MRJP2 Disrupts Cell Membrane Permeability and Cell Wall of P. larvae

The bacterial cell membrane is the main target for many bactericides. The cytoplasmic membrane, mainly composed of a phospholipid bilayer and proteins, plays essential roles in maintaining cellular homeostasis, and provides a barrier to control the input and output of cellular components. It is essential for energy production via both respiration and cell division, and its damage can cause cell inactivation and/or death [54]. Here, the significantly increased intracellular Ca^2+^ concentration in *P. larvae* treated by N-MRJP2 compared to the control by both ToF-SIMS and Fluo 3-AM analyze indicates serious and irreversible occurrence of cytoplasmic membrane damage, thereby resulting in the elevation of intracellular Ca^2+^ level by an influx of the external Ca^2+^. Moreover, the increased release of UV-absorbing material further demonstrates the disruption of cell membrane that leads to the leakage of intracellular components, such as RNA and DNA [55]. The observed altered membrane permeability gives a possibility that the cell membrane of *P. larvae* is one of the potential antibacterial targets for N-MRJP2.

An elevated intracellular Ca^2+^ level can result in an increase in mitochondrial reactive oxygen species (ROS), which then leads to oxidative damage to cells, disturbance of the membrane lipid composition and energy metabolism, and ultimately inducement of apoptosis or cell death [56]. For instance, the appearance of YwfI in the treated group, an anaerobic protein marker that is only induced under anaerobic conditions, suggests that aerobic respiration may be inhibited after damage of the cell membrane of *P. larvae* in the presence of N-MRJP2 [57]. This is also reflected by the down-regulated proteins related to electron transport and ATP synthesis in the treated group relative to the control. Hence, the inhibitory activity of N-MRJP2 on *P. larvae* is likely driven by the disruption of the cellular membrane system and hindering energy generation.

Chemotaxis is important for bacteria to move toward the highest concentration of food molecules for nutrition, or to flee from adverse stimuli. McpC, a chemoreceptor, plays vital roles in sensing chemical gradients. The response to positive stimulus, such as the supplement of carbohydrates, is primarily McpC-mediated. A null mutant of *mcpc* does not respond to the addition of D-glucose in *B. subtilis* [58]. Here, the consistent down-regulated McpC in proteome data and confirmed by western blot analysis indicates that the response to food stimulation may be impaired in the N-MRJP2 treated *P. larvae*. Hence, shortage of nutrients occurs and the growth of the bacteria is blocked.

The peptidoglycan cell wall of bacteria is essential in providing most bacteria with a well-defined cell-shape to resist intracellular and extracellular pressures. The biosynthetic pathway of peptidoglycan and the proteins required for its assembly are the targets for many antibacterial agents [59]. Here, the down-regulated proteins involved in peptidoglycan biosynthesis pathway of *P. larvae* treated by N-MRJP2, such as pbpF, murG, implies that the N-MRJP2 may hinder the peptidoglycan biosynthesis and then block cell wall synthesis. PbpF is a member of penicillin binding proteins that is critical for peptidoglycan synthesis and in sporulation of *B. subtilis* [42], is also involved in the formation of the spore germ cell wall, and its mutant results in a severe sporulation defect of *B. subtilis* [60]. MurG is an N-acetylglucosamine transferase responsible for the last step in the synthesis of peptidoglycan precursor lipid II, a precursor molecule in the synthesis of the cell wall of bacteria. The mutation of *MurG* usually leads to a morphology defect of *B. subtilis*, exhibiting a twisted or undulating curved appearance. Moreover, dacF is related to the synthesis of spore peptidoglycan, and the regulation of the cross-linking degree of spore peptidoglycan [61]. Therefore, the cell wall of *P. larvae* is another possible target for the N-MRJP2 to accomplish its antibacterial activity.

The bacterial life cycle in infected larvae usually goes into two phases. In the early stage, *P. larvae* are non-invasive and live on the larval sugar diet, such as glucose and fructose. The non-invasive phase is followed by penetration of the midgut epithelium and subsequent invasion of the haemocoel via the paracellular route by sequentially destroying the peritrophic membrane, cell-cell junctions, the extracellular matrix [62]. Secreted extracellular proteases, which are able to degrade antimicrobial peptides and might neutralize the local immune response, are likely to be involved in this process. Here, the down regulation of protease (protease PrsW) after the treatment of N-MRJP2 on *P. larvae* may reduce the neutralization of antimicrobial materials, which is, in turn, to block the disruption of the epithelial barrier integrity and prevent the invasion of *P. larvae* to the honeybee larval haemocoel.

Many antibacterial active proteins, including disease-resistant proteins, are glycosylated [63]. The fact that only N-MRJP2 can kill *P. larvae* is in accordance with the notion that in some cases, only full-length or native proteins have a biological effect in cell culture or animal studies [64]. The attachment of N-glycans to the peptide backbones that prevent pepsin hydrolysis of proteins can increase the stability of MRJP2, or potentially act as a decoy receptor for pathogens during the immune and antibacterial response since glycans can be specific binding sites for a variety of viruses, bacteria, and parasites [65]. The oligosaccharide sequences are also more likely to mediate specific recognition events or they provide modulation of some biological processes, such as protein targeting, cell-matrix interactions or cell-cell interactions during the N-MRJP2 treatment [65]. Gut microbial communities of honeybees are erratic and vary with age, caste and season [66]. The tending activities from nurse bees may lead to a bacterial accumulation in larval guts [67]. The documented different glycopatterns of N-MRJP2 [29] may reflect the specific requirement for protection against different pathogenic infections and the varying microflora which colonize the gut of honeybee larvae [68].

In general, the antimicrobial activity towards *P. larvae* of ERIC II is associated with the increased membrane permeability and the blocking of the cell wall biosynthesis. This is driven by impaired cell function [69], leaked cell contents, altered ion transport, inhibited respiration [70], and hindered chemotaxis to positive stimuli. Finally, the cell division is restrained, and cell death is induced by insufficient energy and nutritional resource supplementation.

## 5. Conclusions

Our data, for the first time, reveal the passive immunization activity of RJ on honeybee larvae and the molecular underpinning of antibacterial activity of natural based N-MRJP2 on *P. larvae*. RJ is vital for the immune response of honeybee larvae, especially for small larvae with an immature innate system. N-MRJP2 treatment can disturb the cell membrane system, hinder the biosynthesis of the cell wall, inhibit aerobic respiration, and prevent the chemotaxis to nutritional resources, thereby restraining cell division and inducing cell death of *P. larvae*. The reported data add evidence on RJ biological functions, partially for the development and application of natural antibiotics from RJ on *P. larvae* and other Gram-positive bacteria.

## Data Availability

Data available in a publicly accessible repository.

## Supplementary Materials

Supplementary Materials can be found at https://www.mdpi.com/2218-273X/11/1/64/s1. Figure S1: Concentration dependent inhibitory effect of royal jelly aqueous solution on bacteria, Table S1: Membrane protein identification of *Paenibacillus larvae* before (sheet 1) and after (sheet 2) treated with N-glycosylated major royal jelly protein 2. The mass spectrometry proteomics data have been deposited to the ProteomeXchange Consortium (http://proteomecentral.proteomexchange.org) via the iProX partner repository with the dataset identifier PXD019657.
