# Supplementary material for: Mechanistic Insight into Royal Protein Inhibiting the Gram-Positive Bacteria
Source: Biomolecules. 2021 Jan 6;11(1):64. doi: 10.3390/biom11010064 (PMC7825125; doi:10.3390/biom11010064)
Supplement: Supplementary file 1 [file biomolecules-11-00064-s001.zip › Figure S1.pdf]

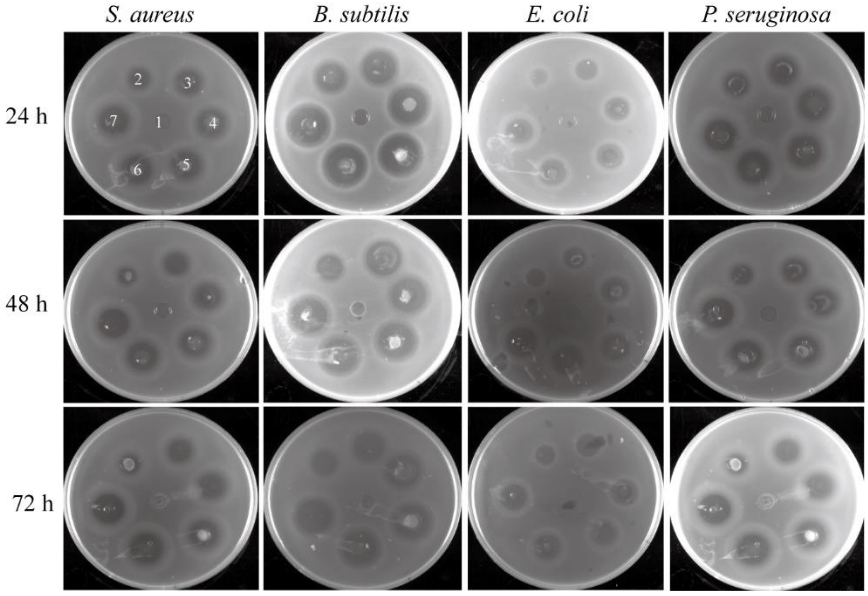

Figure S1. Concentration dependent inhibitory effect of royal jelly aqueous solution on bacteria. Royal jelly was collected at 24, 48 and 72 h after the honeybee larvae transferred into the queen cells. Royal jelly was mixed with distilled water and then 100 µl of each was applied to *Staphylococcus aureus* (*S. Aureus*), *Bacillus subtilis* (*B. Subtilis*), *Escherichia coli* (*E. Coli*), and *Pseudomonas aeruginosa* (*P. Aeruginosa*) using oxford cup, respectively. “1”, blank control (double-distilled water); “2”, 5% (w/w); “3”, 10% (w/w); “4”, 20% (w/w); “5”, 40% (w/w); “6”, 60% (w/w); “7”, 80% (w/w).
